# Sponge chemical defenses are a possible mechanism for increasing sponge abundance on reefs in Zanzibar

**DOI:** 10.1371/journal.pone.0197617

**Published:** 2018-06-20

**Authors:** Stephanie B. Helber, Dieuwke J. J. Hoeijmakers, Christopher A. Muhando, Sven Rohde, Peter J. Schupp

**Affiliations:** 1 Leibniz Centre for Tropical Marine Research (ZMT), Bremen, Germany; 2 Institute for Chemistry and Biology of the Marine Environment (ICBM), Carl-von-Ossietzky University Oldenburg, Wilhelmshaven, Germany; 3 Institute of Marine Sciences (IMS), University of Dar es Salaam, Stonetown, Zanzibar, Tanzania; 4 Helmholtz Institute for Functional Marine Biodiversity at the University of Oldenburg, Oldenburg, Germany; University of Genova, ITALY

## Abstract

Coral reefs are experiencing increasing anthropogenic impacts that result in substantial declines of reef-building corals and a change of community structure towards other benthic invertebrates or macroalgae. Reefs around Zanzibar are exposed to untreated sewage and runoff from the main city Stonetown. At many of these sites, sponge cover has increased over the last years. Sponges are one of the top spatial competitors on reefs worldwide. Their success is, in part, dependent on their strong chemical defenses against predators, microbial attacks and other sessile benthic competitors. This is the first study that investigates the bioactive properties of sponge species in the Western Indian Ocean region. Crude extracts of the ten most dominant sponge species were assessed for their chemical defenses against 35 bacterial strains (nine known as marine pathogens) using disc diffusion assays and general cytotoxic activities were assessed with brine shrimp lethality assays. The three chemically most active sponge species were additionally tested for their allelopathic properties against the scleractinian coral competitor *Porites* sp.. The antimicrobial assays revealed that all tested sponge extracts had strong antimicrobial properties and that the majority (80%) of the tested sponges were equally defended against pathogenic and environmental bacterial strains. Additionally, seven out of ten sponge species exhibited cytotoxic activities in the brine shrimp assay. Moreover, we could also show that the three most bioactive sponge species were able to decrease the photosynthetic performance of the coral symbionts and thus were likely to impair the coral physiology.

## Introduction

Coral reefs worldwide have experienced substantial losses of coral cover and species diversity over the past decades in response to various anthropogenic drivers [1–3]. These declines in coral cover have resulted in shifts of benthic community composition [4–6]. Non reef-building taxa that can cope better with anthropogenic stressors, such as climate change, eutrophication, sedimentation, and disease prevalence, continue to increase in abundance [4,5,7–10]. Additionally, organisms other than hard corals are released from top-down control through overfishing and can undergo uncontrolled growth due to the absence of their predators [10–13]. Sponges are one of the top spatial competitors for reef-building scleractinian corals and their abundance, as well as biomass, on reefs worldwide is steadily increasing [6,14,15]. In the Caribbean, sponge biomass and coverage is now equal to or even exceeding that of corals [10,16–18]. Their great success can be partly explained by their ability to feed on a wide variety of nutritional sources [19], the very low energetic costs of their filter-feeding activities [20] and their strong chemical defenses [18].

Sponges produce the greatest diversity of secondary metabolites among benthic marine organisms [21] with more than 5300 currently described [22]. The primary interest in sponge secondary metabolites has been related to their potential pharmacological activity, but a growing number of studies have started to investigate the ecological functions of these compounds. Their secondary metabolites give sponges the ability to deter predators [23–25], inhibit pathogenic microbes [25–27] and demonstrate competitive dominance towards other sessile benthic organisms [15,28,29].

A crucial factor contributing to the competitive success of sponges is their ability to combat microbial attacks. Many sponges defend their surface from colonization by fouling organisms as well as from potential pathogenic bacteria by producing secondary metabolites with antimicrobial properties [25,30–32]. Marine organisms are constantly exposed to potentially harmful bacteria. In the Indian Ocean bacterial abundances range from 6x 10^4^ ml^-1^ to 2.5x 10^6^ cells ml^-1^ in the surrounding seawater [33–35]. Sponges are additionally exposed to large quantities of microbes passing through their bodies due to their filter feeding activities [20]. Given the exposure of sponges to high numbers of bacteria in the marine environment and the relatively low incidence of infection with diseases, chemical compounds in sponges are likely crucial in providing effective defenses against the invasion of pathogenic microbes after damage or injury [7,25,36–38].

Over the last 20 years the prevalence and severity of marine diseases has increased substantially, particularly impacting reef building corals [5,39,40]. Coral diseases have been correlated with environmental stress caused by human activities and environmental alterations associated with global climate change [39–41]. Diseased corals in the Caribbean were almost exclusively found in anthropogenically impacted areas [42] and some coral diseases are assumed to be caused by human faecal bacteria [43,44]. Sponges on the other hand, seem to be less susceptible to environmental conditions that are stressful for corals or might have better antimicrobial defenses since sponge diseases are much less prevalent [5,7,45].

In addition to their strong defenses against microbial attacks, many sponges are assumed to be competitively superior over other reef organisms [28,46–48]. One of the main factors shaping the community composition of sessile, benthic assemblages is the competition for space [49]. Especially on coral reefs, free substratum space with adequate irradiance for photosynthesis and exposure to food-providing water currents is one of the most limiting resources for benthic organisms [50,51]. The high biodiversity on coral reefs results in high frequency of competitive interactions between sessile organisms of the same and of different species [52,53]. Sponges have not only the ability of rapidly overgrowing benthic reef organisms but they also release chemical compounds that can harm and kill other competitors [15,28,46,48]. Cytotoxic secondary metabolites produced by sponges may be able to inhibit the growth of other competing organisms by impairing their cell division and thus provide sponges with an advantage during competition for space on crowded coral reef substratum [54,55]. The bioactivity, especially the cytotoxicity, of sponge extracts seems to be a good proxy for their ability to overgrow corals in the field [56]. The four most bioactive sponges in the Spermonde Archipelago were also reported to cause necrosis of corals in more than 85% of interactions observed *in situ* [15]. Bioactive compounds are released through tissue contact, via sponge mucus or directly into the surrounding water causing bleaching and tissue necrosis in neighbouring corals thereby reducing their chances of survival [28,46,57]. Several sponges have already been identified to use allelopathy in order to inhibit the growth of other benthic organisms or were even able to cause bleaching and tissue necrosis in neighbouring corals [15,28,46,48,58]. The competitive abilities of sponges may be further enhanced with increasing anthropogenic disturbances that are stressful to corals but tolerable for sponges [59]. In the Western Indian Ocean (WIO) corals were greatly affected by the 1997/1998 El Niño Southern Oscillation (ENSO) resulting in mass coral bleaching and a decline in coral cover [60–62]. This decline of coral species has led to a shift of the ecological balance in favour of other space-competing functional groups especially corallimorpharians [4,63,64]. Additionally, the reefs on Zanzibar´s West Coast are heavily exposed to untreated sewage and runoff from the main city which could represent a potential source for the introduction of a variety of bacteria and pathogens [65,66]. Multiple drainage pipes, some extending up to 55m from the coast along the sea bottom, come from the 2289 septic tanks, and discharge daily 2.2 x 10^6^ l of wastewater [65]. Over the last 12 years an increase of the amount of ^15^N in common benthic organisms, and an increased amount of fecal indicator bacteria (i.e. *Enterococcus* (ENT)) suggest that water quality has deteriorated [65,67–70].

In this study we provide the first evaluation of chemical competitive defenses in Western Indian Ocean (WIO) reef sponges. Our aim was to examine the organic extracts from the most abundant sponge species on reefs around the West Coast of Zanzibar for ecologically significant antimicrobial and cytotoxic activities. Additionally, extracts of the three most active sponge species, *Pseudoceratina* sp., *Callyspongia* sp. and *Haliclona atra*, were tested for their allelopathic properties in field experiments. Corals of the genus *Porites* were chosen for the field experiments since the reefs at Bawe Island are dominated by large monostands of branching and massive *Porites* following the El Niño in 1997/ 1998 and a Crown-of-Thorn Starfish (COTS) outbreak in 2002–2006 [8,71–74]. Furthermore, all three sponge species were observed to grow adjacent or even in between branching *Porites* corals, making the study of interactions among these two organisms ecologically relevant.

## Material and methods

### Ethics statement

This research was completed in accordance with permits issued by the Research Committee of the Zanzibarian Government.

### Study site

The field study was conducted from September to December 2014 at reefs around Bawe Island, Zanzibar (Unguja), Tanzania (S1 Fig). Bawe (06° 09´25.56” S, 39° 08´0.96” E) is located on the west side of the island in the Zanzibar channel, about 7km from the capital Stonetown [71]. The reef at Bawe is heavily influenced by fishing activities and untreated sewage discharge from Stonetown and its harbour [8,65,66,75].

### Collection and extraction procedure

Based on a sponge community survey [76], the ten most abundant sponge species were chosen for the investigation of their antimicrobial and cytotoxic properties. This included *Callyspongia aerizusa*, *Callyspongia* sp., *Haliclona atra*, *Haliclona fascigera*, *Biemna* sp., *Paratetilla* sp., *Pseudoceratina* sp. *Scopalina hapalia*, *Plakortis kenyensis* and *Tetrapocillon minor*. The sponge species were identified by Dr. Nicole de Voogd and vouchers have been registered in the sponge collection of the Naturalis Biodiversity Center in Leiden, Netherlands. Specimens of the selected sponge species were sampled opportunistically by scuba-divers at 10m depth. Replicates (3–5 individuals) of each sponge species were collected and transferred into zip block bags filled with ambient seawater. There was a minimum distance of 20 m between replicates to avoid collection of clones. Samples were immediately transferred in coolers to the laboratory facilities at the Institute of Marine Sciences (IMS, Stonetown). After gently removing dripping water, sponge pieces were weighed to the nearest 0.1 g to determine wet weight (WW), cut into small pieces and secondary metabolites were extracted three times with 99.9% Ethanol. The extracts were filtered to remove particles and the filtrate was kept at ‐20°C for storage and transport. Samples were filtered again and evaporated under reduced pressure using a rotary evaporator (water bath temperature 35°C) at the laboratories of the ICBM, University of Oldenburg. The crude extracts were transferred into preweighted glass vials and evaporated to complete desiccation with a Speed Vac. Natural extract concentrations were calculated as mg extract per gram of sponge wet weight (see Table 1). Extracts from 3–5 replicate sponge individuals per species were pooled and used for the experiments to get a significant estimate of the response parameters for this population. This has been done in many studies on chemical defense (e.g. [76–80]). All extracts were stored at -20°C until use.

**Table 1:** The most abundant sponge species at Bawe Island, Zanzibar, their percent coverage and natural extract yield. The data for the benthic cover of the different sponge species were obtained by a previous study [76].

Order	Species	Number of Replicates	Extract yield [mg g (WW)^-1^] ^a^	Benthic cover at 10m depth[%]
**Haplosclerida**	*Haliclona fascigera*	5	13.00 (± 6.38)	0.17 (±0.90)
**Haplosclerida**	*Haliclona atra*	3	26.51 (± 4.53)	2.33 (±4.70)
**Haplosclerida**	*Callyspongia aerizusa*	5	19.35 (± 10.14)	0.37 (±1.32)
**Haplosclerida**	*Callyspongia* sp.	3	22.90 (± 2.18)	0.13 (±0.48)
**Verongida**	*Pseudoceratina* sp.	3	31.06 (± 18.63)	0.00 (±0.00)
**Homosclerophorida**	*Plakortis kenyensis*	3	16.80 (± 13.25)	0.01 (±0.03)
**Scopalinida**	*Scopalina hapalia*	3	17.14 (± 5.86)	0.04 (±0.14)
**Poecilosclerida**	*Biemna* sp.	3	18.29 (± 2.84)	2.88 (±4.20)
**Tetractinellida**	*Paratetilla* sp.	3	26.31 (± 3.66)	0.01 (±0.04)
**Poecilosclerida**	*Tetrapocillon minor*	4	18.39 (± 7.37)	0.11 (±0.24)

^a^ Extracts yields in mg extract per g WW (wet weight) are given as the mean of 3–5 extractions (± STD).

### Bacterial panel

The agar disc-diffusion assay was used to assess antibacterial activities of the sponge crude extracts. 35 bacterial strains, representing a wide phylogenetic range (see Table 2), were tested including nine known pathogens associated with marine diseases (*Aurantimonas coralicida*, *Acitenobacter pitiii*, three strains of *Vibrio alginolyticus*, *Vibrio owensii*, two strains of *Vibrio coralliilyticus* and *Vibrio shilonii*). Bacteria were considered pathogens if their closest match was a bacterial strain associated with a marine disease and they further possessed sequence similarities of >98% to known pathogens in the 16S NCBI BLAST database.

**Table 2:** Description of the 35 bacterial strains used in the antimicrobial assay, including nine known pathogens for marine diseases percent similarity indicates how close the bacterial sequence of the isolate is to the closest strain in the NCBI BLAST databank.

No.	Phylum	Class	Family	Accession no. of bacterial isolate	Species (closest NCBI hit)	Accession no. of closest NCBI hit	Similarity of the closest NCBI hit	
1701	*Actinobacteria*	*Actinobacteria*	*Micrococcaceae*	MG551787	*Kocuria halotolerance*	NR_044025	98.888	
1744	*Actinobacteria*	*Actinobacteria*	*Micrococcaceae*	MG551810	*Micrococcus aloeverae*	NR_134088	99.666	
1682	*Actinobacteria*	*Actinobacteria*	*Nocardiaceae*	MG551778	*Rhodococcus corynebacterioides*	NR_119107	99.343	
1656	*Actinobacteria*	*Actinobacteria*	*Streptomycetaceae*	MG551768	*Streptomyces flavoviridis*	NR_041218	100	
1733	*Proteobacteria*	*Alphaproteobacteria*	*Rhodobacteraceae*		*Loktanella pyoseonensis*	NR_115100	98.684	
1636	*Bacteroidetes*	*Flavobacteria*	*Flavobacteriaceae*	MG551762	*Aquimarina gracilis*	NR_113781	99.302	
1686	*Firmicutes*	*Bacilli*	*Bacillales Family XII*. *Incertae*	MG551781	*Exiguobacterium profundum*	NR_043204	99.549	
1694	*Proteobacteria*	*Alphaproteobacteria*	*Rhodobacteraceae*	MG551784	*Paracoccus zeaxanthinifaciens*	NR_025218	99.892	
1754	*Proteobacteria*	*Gammaproteobacteria*	*Rhodobacteraceae*	MG551813	*Ruegeria areniliticus*	NR_109635	99.678	
1668	*Proteobacteria*	*Alphaproteobacteria*	*Rhodobacteraceae*	MG551772	*Ruegeria areniliticus*	NR_109635	97.439	
1792	*Proteobacteria*	*Alphaproteobacteria*	*Rhodobacteraceae*	MG551832	*Pseudovibrio denitrificans*	NR_113946	99.784	
1721	*Proteobacteria*	*Gammaproteobacteria*	*Alteromonadaceae*	MG551801	*Microbulbifer variabilis*	NR_041021	99.78	
1633	*Proteobacteria*	*Gammaproteobacteria*	*Pseudoalteromonadaceae*	MG551852	*Pseudoalteromonas phenolica*	NR_113299	99.785	
1783	*Proteobacteria*	*Gammaproteobacteria*	*Pseudoalteromonadaceae*	MG551825	*Pseudoalteromonas piscicida*	NR_114190	99.251	
1810	*Proteobacteria*	*Gammaproteobacteria*	*Enterobacteriaceae*	MG551842	*Pantoea eucrina*	NR_116246	99.299	
1703	*Proteobacteria*	*Gammaproteobacteria*	*Pseudoalteromonadaceae*	MG551789	*Pseudomonas pseudoalcaligenes*	NR_037000	98.168	
1652	*Proteobacteria*	*Gammaproteobacteria*	*Vibrionaceae*	MG551766	*Vibrio maritimus*	NR_117551	98.28	
1809	*Proteobacteria*	*Alphaproteobacteria*	*Rhodobacteraceae*	MG551841	*Ruegeria areniliticus*	NR_109635	98.072	
1767	*Proteobacteria*	*Gammaproteobacteria*	*Pseudoalteromonadaceae*	MG551817	*Pseudomonas luteoviolacea*	NR_026221	99.466	
1727	*Actinobacteria*	*Actinobacteria*	*Micrococcaceae*	MG551804	*Kocuria sediminis*	NR_118222	99.024	
1726	*Proteobacteria*	*Gammaproteobacteria*	*Pseudoalteromonadaceae*	MG551803	*Pseudoalteromonas piscicida*	NR_114190	99.679	
1722	*Proteobacteria*	*Gammaproteobacteria*	*Halomonadaceae*	MG551802	*Halomonas aquamarina*	NR_042063	99.465	
1334	*Proteobacteria*	*Gammaproteobacteria*	*Alteromonadaceae*	MG711595	*Aliagarivorans marinus*	NR_044585	98	
1348	*Proteobacteria*	*Gammaproteobacteria*	*Vibrionaceae*	MG711594	*Vibrio maritimus*	NR_117551	98	
0852	*Proteobacteria*	*Gammaproteobacteria*	*Moraxellaceae*	MG551849	*Acitenobacter soli*	NR_044454	99	
1659	*Proteobacteria*	*Gammaproteobacteria*	*Pseudoalteromonadaceae*	MG551769	*Pseudomonas luteoviolacea*	NR_114237	99.678	
**Known pathogens for marine diseases**								**Info about pathogens**
1678	*Proteobacteria*	*Gammaproteobacteria*	*Moraxellaceae*	MG551777	*Acinetobacter pitii*	NR_117930	99.663	Fish pathogen [82] and human pathogen (Pneumonia; [83])
1621	*Proteobacteria*	*Gammaproteobacteria*	*Vibrionaceae*	MG551759	*Vibrio alginolyticus*	NR_113781	99.302	Marine pathogen, associated with several diseases in fish and shrimp [84–88]
1645	*Proteobacteria*	*Gammaproteobacteria*	*Vibrionaceae*	MG551765	*Vibrio owensii*	NR_117424	99.569	Tissue loss disease "Montipora White Syndrome" in the Hawaiian reef coral *Montipora capitata* [89]
1675	*Proteobacteria*	*Gammaproteobacteria*	*Vibrionaceae*	MG551775	*Vibrio alginolyticus*	NR_113781	98.783	Marine pathogen, associated with several diseases in fish and shrimp [84–88]
1761	*Proteobacteria*	*Gammaproteobacteria*	*Vibrionaceae*	MG551816	*Vibrio coralliitycus*	NR_117892	99.57	Bacterial bleaching and rapid tissue destruction [90–92]
1644	*Proteobacteria*	*Gammaproteobacteria*	*Vibrionaceae*	MG551764	*Vibrio alginolyticus*	NR_113781	99.785	Marine pathogen, associated with several diseases in fish and shrimp [84–88]
WHV0001	*Proteobacteria*	*Alphaproteobacteria*	*Aurantimonadaceae*		*Aurantimonas coralicida*	AY065627	DSMZ, Germany	White plague type II disease [93]
WHV0002	*Proteobacteria*	*Gammaproteobacteria*	*Vibrionaceae*		*Vibrio shilonii*	ATCC BAA-91	DSMZ, Germany	Bacterial bleaching [94,95]
WHV0003	*Proteobacteria*	*Gammaproteobacteria*	*Vibrionaceae*		*Vibrio coralliilyticus*	AJ440005	DSMZ, Germany	Bacterial bleaching and rapid tissue destruction [90–92]

The bacterial strains were formerly isolated from the crustose coralline alga *Hydrolithon reinboldii* and from two sponges, namely *Rhabdastrella globostellata* (no.1334 and 1348) and *Pseudoceratina* sp. (no.0852) from Guam, except for three marine pathogens (WHV0001, WHV0002 and WHV0003) that were ordered from the Deutsche Sammlung von Mikroorganismen und Zellkulturen GmbH, DMSZ, Braunschweig, Germany. The isolation, conservation and identification of the various bacterial strains have been performed by our colleagues [25,81]. The comparison of the sequence data from the isolated strains with sequences in the NCBI BLAST database was according to methods described by our colleagues [81].

### Antimicrobial assay

Bacterial strains were grown in liquid marine broth medium for 24 hours at 25°C before each experiment. Crude extracts of the different sponge species were dissolved in aliquots of ethanol at natural concentrations (see Table 1). 15 μl of crude extract were added to sterile filter paper (Ø 6 mm, Whatman) and the solvent was allowed to completely evaporate. Control filters were prepared in the same manner with 15 μl of solvent only. Each one of the 196 Marine broth agar plates (1.5% agar, 3.75 g l^-1^ Difco Marine Broth 2216, filtered deionized water) was inoculated with 200 μl of liquid culture of the respective test strains and spread evenly to provide a uniform bacteria lawn. Up to seven extract discs and one solvent control disc per plate were randomly assigned to the different plates and placed on the surface of agar plates with the extract side facing the plate. Three to six replicates of each pooled extract were tested, depending on the abundance of the sponges in the field during collection. Following a 24 h and 48 h incubation period, growth inhibition zones were scored as clear halos around the discs. The radius of the inhibition zone (without disc) was measured to the nearest 0.5 mm.

Permutational multivariate analyses of variance (PERMANOVA) obtaining Monte Carlo p-values (due to too low numbers of permutations) were conducted with the Primer software (Version 6.1.13) and the PERMANOVA+ add-on (Version 1.03). The analyses were used to test for significant activities of sponge extracts and to compare activities against environmental and pathogenic bacteria [96,97]. Results were categorized as no effect (0), weak inhibition (0–1mm), moderate inhibition (>1 to 3mm), strong inhibition (>3 to 7mm) and very strong inhibition (>7 to 15mm) after [98].

### Brine shrimp lethality assay

Brine shrimp (*Artemia salina*) eggs were placed in a hatching tank containing seawater with strong aeration under continuous light at 24°C for 12 hours. Photophilic nauplii were collected approximately 12 hours after hatching. A stock solution of 10mg ml^-1^ was prepared for each pooled extract of the ten sponge species. From the stock solutions 1000 and 100 μl were transferred to individual petri dishes and solvents were allowed to evaporate. The respective amount of ethanol served as control. After the solvents had evaporated, 5 ml of filtered (0.45 μm), autoclaved, seawater was added to each petri dish. The petri dishes were placed on a shaker for 30 minutes to ensure that the extracts dissolved in the seawater. Subsequently, ten brine shrimps were added to each petri dish and the total volume was adjusted to 10 ml resulting in a final extract concentration of 1 mg, 0.1 mg and 0.01 mg sponge crude extract per 10 ml seawater. Larvae were not fed during the experiments as they still rely on their yolk-sac [99] and can survive for up to 48 hours without food [100]. Toxicity was determined after 48 hours (instar III/IV stage) of exposure by counting the surviving nauplii. This time was chosen since most extracts displayed an increasing activity up to 48 hours of exposure [101]. In addition, *Artemia* nauplii have been shown to exhibit their greatest sensitivity to sponge compounds in the second and third instar larval phase [100,102]. Extracts and controls were prepared in triplicates. Larvae were considered dead if no internal or external movement could be observed.

A PERMANOVA obtaining Monte Carlo p-values (due to too low numbers of permutations) was conducted with the Primer software (Version 6.1.13) and the PERMANOVA+ add-on (Version 1.03) to test for significant differences in mortality rates of brine shrimp between the individual sponge extracts and the control (n = 6 for each sponge species and the control).

### Allelopathic activities of sponge extracts

Field experiments determined the allelopathic activities of sponge extracts under natural conditions. Assays with sponge extracts incorporated into phytagel were adapted from past studies of sponge overgrowth experiments and sponge-coral interactions [28,48]. We incorporated sponge extracts of *Pseudoceratina* sp., *Callyspongia* sp. and *Haliclona atra* at natural concentrations into phytagel strips and placed them in contact around branches of the corals of the genus *Porites*. Treatment phytagel strips with natural gravimetric concentrations of sponge extracts and control phytagel strips with only the extract solvent were prepared as described in [48]. Ethanol was used as solvent to extract allelopathic compounds of *Pseudoceratina* sp., *Callyspongia* sp. and *H*. *atra*. The phytagel was poured into a rectangular mold backed with a gaze. The phytagel mixture was allowed to cool and harden onto the gaze before being cut into strips with a small square patch of phytagel in the center of each strip. The gaze strips were transported in zip block bags on the same day to the reef of Bawe and fixed with rubber bands around coral branches of different *Porites* individuals at depths of 4–6 m. Six colonies that displayed no signs of bleaching were chosen for the experiment and marked with numbered tags.

For each sponge extract, one control (solvent only) and one treatment (with extract) strip was attached to adjacent coral branches. For extracts of *H*. *atra* and *Pseudoceratina* sp. six replicate coral colonies, and for the extracts of *Callyspongia* sp. four replicate colonies were used (the remaining two strips were lost during the dive limiting this to 4 replicates). Coral colonies were from the same fringing reef and treatment occurred between 13:00 and 15:00 h. Measurements of the photosynthetic efficiency of the zooxanthellae were taken by a diving PAM fluorometer (Walz, Germany) after 16–18 hours, before sunrise of the next day, in order to estimate the maximum quantum yield of the dark-adapted coral tissue. Under each phytagel patch, 6–8 measurements were taken, as well as from coral branches of the same colony that had neither treatment nor control phytagel strips attached to account for effects of shading, abrasion and the physical presence of the gel. For all readings of the PAM fluorometer, a clip was used that kept the probe at a distance of 1 cm from the coral surface. PAM fluorometry is a non-invasive technique and even though the use of the maximum photosynthetic yield as a proxy for intra- and interspecific comparisons of coral health under different environmental conditions is contentious, comparisons of coral tissue from the same coral branch are accepted [103,104]. Permutational multivariate analyses of variance (PERMANOVA) were conducted with the Primer software (Version 6.1.13) and the PERMANOVA+ add-on (Version 1.03). The analysis was used to test for significant differences of the effects of sponge extracts and for the effects of the phytagel with the solvent [96,97].

## Results

### Antimicrobial assay

**Fig 1:**
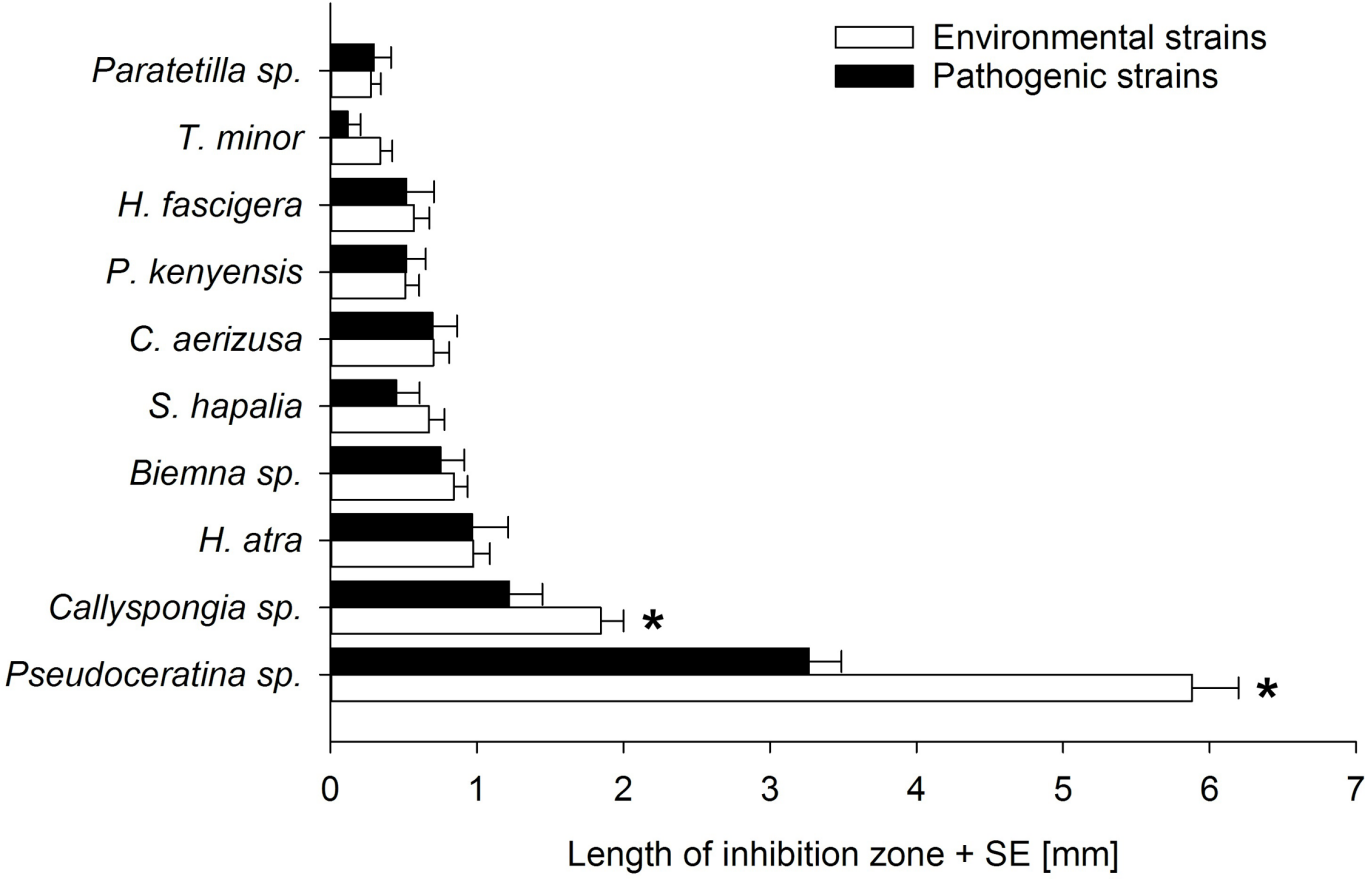
Length of inhibition zones (mean radius, mm + SE) for environmental and pathogenic bacterial strains. Bacterial inhibition by sponge crude extracts for environmental and pathogenic bacterial strains were compared. * indicates a significant difference between the inhibition of environmental vs. pathogenic bacterial strains (PERMANOVA, p < 0.05).

All tested sponge extracts showed antimicrobial activity while solvent control discs never inhibited bacterial growth (Table 3). Antimicrobial effects of the extracts varied widely with respect to the bacterial strains. The extract of *Pseudoceratina* sp. had the strongest antimicrobial activity in terms of the size of the inhibition zones, as well as the number of bacteria inhibited (inhibited all strains). Extracts of *Callyspongia* sp. and *Haliclona atra* revealed the second and third most antibacterial activity (71% and 49% of all strains inhibited, respectively; Table 3). In contrast, *Paratetilla* sp. and *Tetrapocillon minor* were the least chemically defended species inhibiting only 11% of the bacterial strains and also showing the smallest inhibition zones. The other five sponge species displayed all moderate antibacterial activity with 23–34% of the bacterial strains inhibited (Table 3). When comparing the activity of the different sponge extracts against environmental and pathogenic bacterial strains, most sponge species were equally defended against potential pathogens. Only *Pseudoceratina* sp. and *Callyspongia* sp. were better defended against environmental bacteria with respect to the size of the inhibition zones (Fig 1; PERMANOVA, p < 0.05, S1 Table). Detailed results of the disc diffusion assays are presented in S2 Table.

**Table 3:** Degree of antimicrobial activity by crude extracts of ten sponge species.

Sponge species	Number of bacterial strains inhibited (total 35)				
	Weak	Moderate	Strong	Very strong	Sum of strains inhibited (% active)
***T*. *minor***	0	4	0	0	4 (11%)
***Paratetilla* sp.**	0	4	0	0	4 (11%)
***H*. *fascigera***	1	7	0	0	8 (23%)
***P*. *kenyensis***	1	8	0	0	9 (26%)
***S*. *hapalia***	1	6	2	0	9 (26%)
***C*. *aerizusa***	2	8	1	0	11 (31%)
***Biemna* sp.**	3	9	0	0	12 (34%)
***H*. *atra***	8	7	2	0	17 (49%)
***Callyspongia* sp.**	2	18	5	0	25 (71%)
***Pseudoceratina* sp.**	2	6	19	9	35 (100%)

Radius of inhibition zone: 0 no effect; >0–1mm: weak inhibition; >1–3mm: moderate inhibition; >3–7mm: strong inhibition; >7–15mm: very strong inhibition (after Lippert et al. 2003).

S2 Fig shows the antimicrobial activities of the tested sponge species against all *Vibrio* species (environmental as well as pathogenic strains). We choose to display the antimicrobial activities of all sponge species against *Vibrio* spp. because many species of this genus have been recognized as significant pathogens for marine organisms, including sponges [105–109].

### Brine shrimp lethality assay

**Fig 2:**
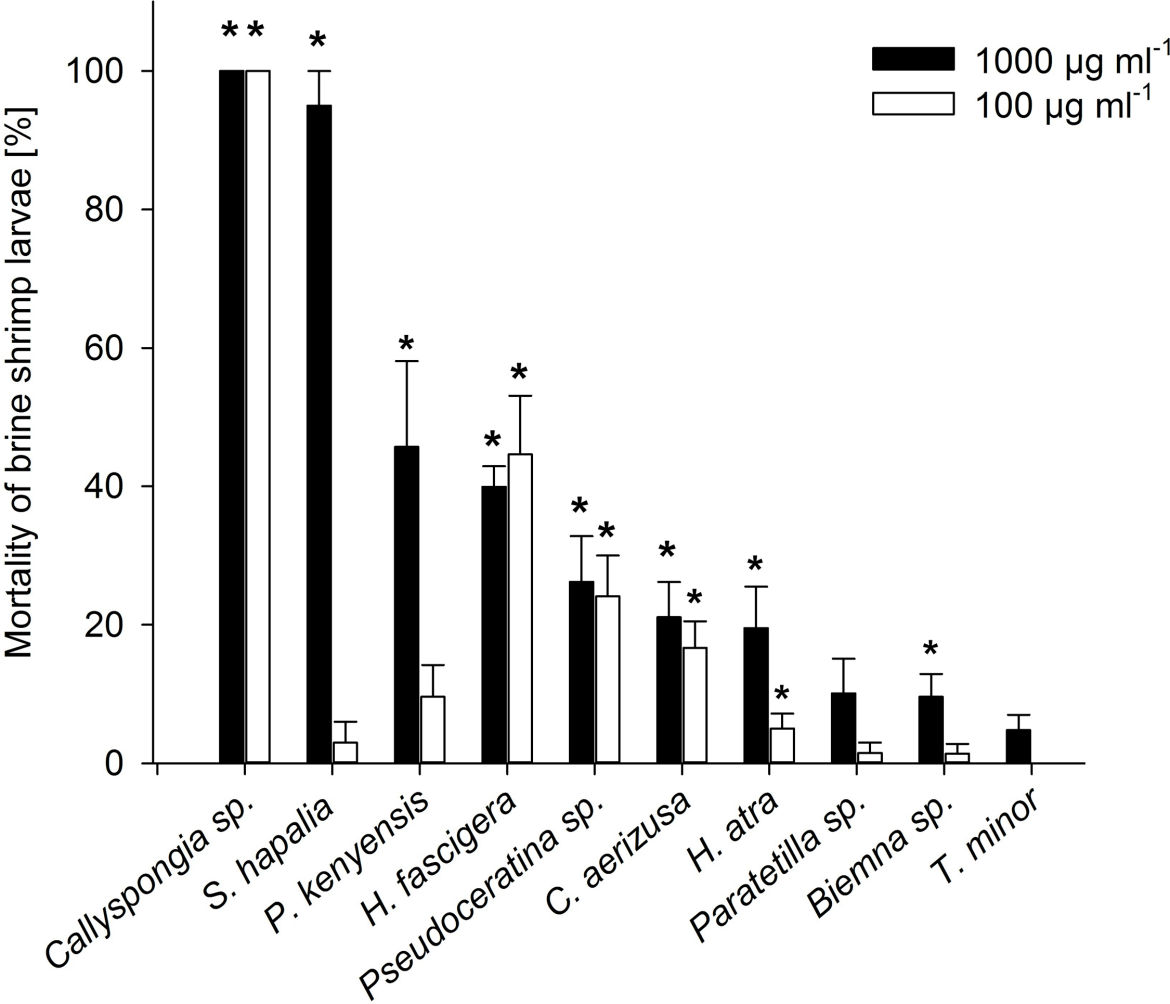
Mortality rates (+ SE) of the brine shrimp larvae in the lethality assay. The mortality rates of the brine shrimp larvae are displayed in response to exposure to the different sponge crude extract concentrations at 1000μg ml^-1^ and 100μg ml^-1^ after 48 hours (mean + SE, n = 6). * indicates a significant mortality rate compared to control (PERMANOVA, p < 0.05).

The results of the brine shrimp bioassay showed that seven out of the ten sponge species contained cytotoxic compounds (Fig 2; PERMANOVA, p < 0.05, S3 Table). The highest lethality was found for extracts of *Callyspongia* sp. at both concentrations, which exhibited *Artemia* mortality rates of 100% after just 12 hours (data not shown). *Scopalina hapalia* caused high mortality with 95% dead nauplii at a concentration of 1000 μg ml^-1^, but only non-significant mortality (5%) at a concentration of 100μg ml^-1^. *H*. *fascigera*, *Pseudoceratina* sp., *C*. *aerizusa*, *H*. *atra* and *P*. *kenyensis* all showed moderate lethality rates between 10.1% and 45% at the two test concentrations. No significant mortality rates were obtained from extracts of *Biemna* sp., *Paratetilla* sp. and *T*. *minor*. No mortality of *Artemia* larvae could be detected in controls. Detailed results of the brine shrimp assays can be found in S4 Table.

### Allelopathic activities of sponge extracts

**Fig 3:**
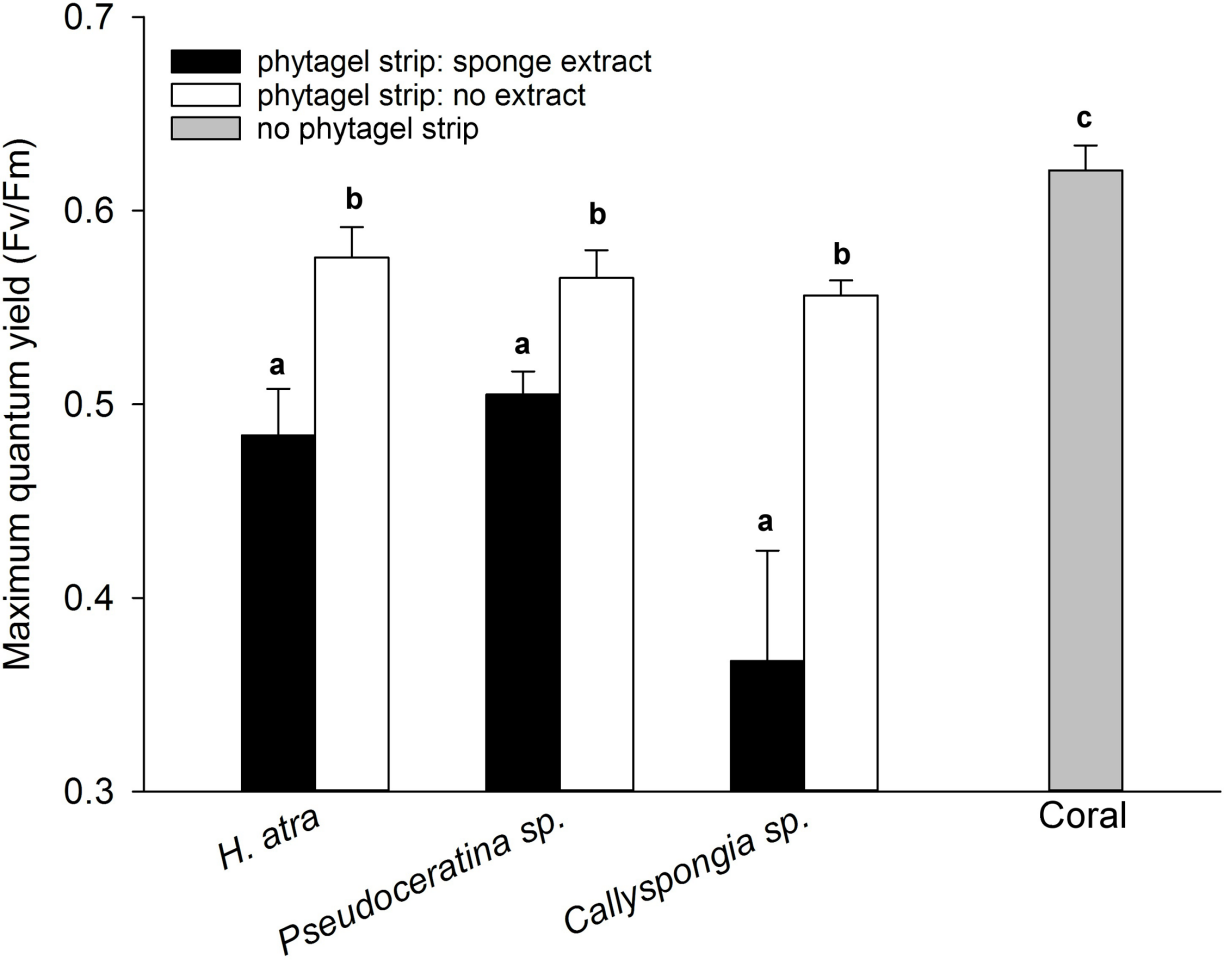
*In situ* allelopathic effects of sponge extracts on the photosynthetic yield of a branching *Porites* coral. Phytagel strips containing natural concentrations of sponge secondary metabolites reduced the maximum photosynthetic quantum yield (bars) of the symbiotic algae (zooxanthellae) in a branching *Porites* coral after 16–18 h of exposure (mean + SE, n = 6, except *Callyspongia* sp., n = 4). Letters indicate significant differences between treatment, control strips and unexposed coral tissue (control coral).

Effects of crude extracts from three Zanzibarian sponge species on the branching coral *Porites* sp. are presented in Fig 3. PAM readings were taken under each sponge extract and control gel band. Control phytagel strips had significant effects on the maximum photosynthetic yield of corals. Nonetheless, phytagel strips with sponge extracts exhibited stronger impairments on the corals photosynthetic performance and were also significantly different from the control strips. The extracts of all three investigated sponges, *H*. *atra*, *Callyspongia* sp. and *Pseudoceratina* sp., showed significant effects on the photosynthetic yield of branching *Porites* corals (PERMANOVA, p = 0.0001, df = 2). Metabolites produced by *Callyspongia* sp. had the most pronounced effect on the photosynthetic yield, decreasing the maximum quantum yield by 41% (PERMANOVA, p = 0.0039, t = 5.6076, df = 3). Extracts of *H*. *atra* and *Pseudoceratina* sp. had comparable negative effects on the effective quantum yield, reducing it by 22% and 19% each (PERMANOVA, p = 0.0014, t = 5.1455, df = 5 for *H*. *atra* and p = 0.0018, t = 6.1752, df = 5 for *Pseudoceratina* sp.). Detailed results of the effects of the different sponge extracts on the maximum quantum yields of individual *Porites* corals are presented in S5 Table.

Sponge metabolites in the gel did not only affect the maximum photosynthetic yield of the *Porites* corals, but caused also bleaching of the underlying coral tissue. Coral tissue exposed to the metabolites of the extract of *Callyspongia* sp. and *H*. *atra* showed the most visibly bleaching.

## Discussion

Sponges are regarded as the richest source of secondary metabolites with diverse bioactive properties, contributing to nearly 40% of all marine biomedical compounds discovered [110,111]. This study is the first that investigated the chemical ecology of sponges from East-Africa, revealing high bioactivity with regards to antimicrobial, cytotoxic and allelopathic effects.

### Antimicrobial assay

All investigated sponge species exhibited antimicrobial activity in varying degrees depending on the number of inhibited test strains (11% up to 100% of strains inhibited). These results are in accordance with other studies examining the antimicrobial activity of sponges in which all sponge species from the Indian Ocean [112,113] and Antarctica [98] displayed antimicrobial activity. All sponges, except for *Pseudoceratina* sp. and *Callyspongia* sp., were equally active against known coral pathogens as well as against bacteria encountered in their environment. However, both of them displayed also moderate to high antimicrobial activities against pathogens, including several *Vibrio* strains. In contrast to our study sponges in the Caribbean displayed weaker inhibition rates for seawater bacteria compared to bacteria inhabiting necrotic sponge tissue or known pathogens [114]. However, other than the use of antimicrobial compounds, sponges possess efficient defense mechanisms that recognize pathogens and initiate an immune response. Sponges distinguish between infectious and non-infectious bacteria through molecular responses, receptor molecules and membrane proteins [115–117]. Bacteria associated with sponges are able to produce molecules which act on the sponge cells by inhibiting its immune and apoptotic system [118–121]. Growth inhibition, which was tested in the present study, is only one of the three stages in the colonization process by bacteria or potential pathogens (attachment, growth and swarming). Secondary metabolites of sponges might interfere with each of the three different colonization stages [27,32].

The strong activity against the variety of bacterial strains exhibited by *Pseudoceratina* sp. in this study indicates that this sponge species produces broad-spectrum antimicrobial compounds. Sponges of the genus *Pseudoceratina* contain bromotyrosine alkaloids and sterols of the aplystane type which possess cytotoxic [122,123], antimicrobial [124,125] as well as anti-HIV [126] and antimalarial [127] activities. Purealin C and its derivatives, which have been isolated from another *Pseudoceratina* species, also exhibit broadspectrum antimicrobial activities [128]. The two *Callyspongia* sponges displayed strong to moderate antimicrobial activity. *Callyspongia* species possess a variety of bioactive secondary metabolites, including Siphondiol [129], Akaterpin [130] and Utenine [131] displaying antimicrobial properties. The third most active sponge genus was *Haliclona*, with *H*. *atra* and *H*. *fascigera* inhibiting 17 (47%) and eight (22%) of the tested bacterial strains, respectively. *Haliclona* spp. produce Manzamine alkaloids with antitumor and antimalarial activities [132,133], as well as the antifungal and the antimicrobial compounds Papuamine, Haliclonadiamide and Halaminoles [134,135]. Additionally, extracts from several Indo-Pacific *Haliclona* species inhibited various bacterial strains, emphasizing the overall antimicrobial activity of this genus [112,136,137]. However, there are also some *Haliclona* species which possess weak or no antimicrobial activity [98,138,139]. The variability in sponge secondary metabolites of the same genus, or even of the same species, is not uncommon as their production is influenced by genetic traits as well as environmental factors and sponge-associated microbes [98,140–144]. *Biemna* sp. displayed moderate antimicrobial activity, inhibiting 12 (33%) of the tested bacterial strains. Sponges of the genus *Biemna* are a source for bioactive compounds with antimicrobial as well as cytotoxic activities, which include pyridoacridines (e.g. Labuanine), steroids (e.g. Ehrenasterol and Biemnasterol) as well as polycyclic alkaloids (e.g. Biemnadin, Hydroxyascididemin and Netamines; [136,137,145]). The sponges *Plakortis kenyensis* and *Scopalina hapalia* were active against nine (25%) of the examined bacterial strains. No reports of secondary metabolites of *S*. *hapalia* were found in the literature. The most prominent antimicrobial compounds isolated from plakinid sponges are Plakinidones, Plakortide, Manzamenone and Plakortin, which showed activity against several bacteria including *Staphylococcus aureus*, *Escherichia coli* and *Bacillus subtilis *[146–150]. No bioactive properties have been reported for the sponges *Paratetilla* sp. and *Tetrapocillon minor*. There is often no correlation between the antimicrobial activity of the sponge extract and the epibacterial abundance in sponges or ascidians [151,152]. Therefore, it would be interesting to test all colonization stages with the extracts of sponges from Zanzibar since *T*. *minor* was a sponge that inhibited the growth of only 11% of the tested strains but had always a very clean and smooth surface (personal observation). Instead of producing antimicrobial compounds, sponges also attract bacteria to their surfaces that repel other biofilm-forming bacteria and thus maintain a clean and smooth surface [153].

These examples also highlight one of the problems when evaluating sponge antimicrobial activities from the literature. Many of the antimicrobial activities reported for sponges used human pathogenic bacteria and not environmental bacteria to which the sponges are exposed to and did not test marine pathogens which pose a potential risk to the sponge holobiont. Our study on the contrary is one of a few that focused on marine environmental and marine pathogen bacteria to evaluate sponge antimicrobial defenses.

There is still a lack of knowledge about the main cause of sponge diseases and the role of pathogenic bacteria [45,154,155] which is why in the present study we utilised pathogenic bacteria known to cause diseases in corals and other marine organisms. Marine pathogens seem to affect not only one host (e.g. corals) but seem to be able to cause diseases also in other hosts if conditions are opportune. For example, cyanobacteria, which were associated with coral diseases, also replaced the *Synechococcus/ Prochlorococcus* clades in sponges affected by sponge orange band disease [154]. Environmental perturbations including urban runoff, nutrient enrichment, anthropogenic pollution and especially increase in temperature are linked to disease outbreaks in marine invertebrates [155–157]. The brown lesion disease in sponges is most likely caused by terrestrial pathogens used in pest control management of insects and fungi [158]. The high prevalence of antimicrobial defenses in the investigated sponges suggests that pathogens are a common threat to sponges on Zanzibar’s reefs.

The coral reefs surrounding Stonetown are especially exposed to untreated sewage and runoff from the main city [65,66]. Human fecal bacteria in wastewater are the etiological agent of white pox and black band disease in corals [43,44]. Even if wastewater does not harbour etiological agents for diseases of benthic invertebrates, the discharge of sewage introduces many opportunistic microbial taxa, such as e.g. *Vibrionaceae* and *Rhodobacteraceae*, that can alter the microbial community in coral as well as sponge holobionts resulting in the onset of disease [90,106–108,159,160]. Nutrient enrichment, especially increase in nitrogen, can facilitate the spread of diseases in corals because pathogens are normally nitrogen limited [39,161,162]. The reef around Bawe Island has higher phosphate and nitrate concentrations compared to other reefs around Zanzibar, which could help to increase pathogen fitness and virulence [66]. Around Bawe only a few cases of sponge diseases have been reported (e.g. necrotic tissue spots in *H*. *fascigera*; [161]), indicating that sponge antimicrobial defenses seem to be efficient in defending sponges from pathogens. The high antimicrobial activity of the investigated extracts might be a response of the sponges to a high prevalence of potential pathogens.

### Brine shrimp lethality assay

The brine shrimp lethality assay is an easy, rapid and inexpensive bioassay to test for the general toxicity of organisms. Brine shrimp are highly sensitive organisms in regard to sponge crude extracts [163,164]. Additionally, there is a strong relationship between the toxicity of brine shrimp assays and the potential anti-tumor activities of extracts [101,165].

In the present study, seven (70%) of the tested sponge species showed cytotoxicity in the brine shrimp assay. In other studies, 59–75% of sponge species also showed cytotoxic activities in brine shrimp or other cytotoxicity assays in line with our results [164–166]. Extracts from all four sponges within the order Haplosclerida displayed significant cytotoxic activities in the brine shrimp assay with *Callyspongia sp*. showing the highest cytotoxic activity followed by the two *Haliclona* species. Their cytotoxic activities were comparable to other Haplosclerida species reported in the literature [167–169]. Significant cytotoxic activities were also detected in extracts of *S*. *hapalia* and *P*. *kenyensis* at the highest test concentration, which is again in line in literature reports [170–172]. The moderate cytotoxic activity of *Pseudoceratina* sp. can most likely be ascribed to bioactive bromotyrosine derivatives in the crude extract [172–175], while the lack of activity in *Biemna* sp. was contrary to previous studies [172,176–179].

Toxic sponge compounds are able to rapidly kill cells of competitors through apoptosis, autophagocytosis and necrosis [54,55,180], thereby inhibiting the growth of other space competing organisms. Allelochemicals with cytotoxic properties have the ability of inhibiting metabolic processes in various coral species as well as killing live coral tissue within a few days [46,181]. Other functions of cytotoxic compounds include the inhibition of photosynthesis in the corals symbiotic zooxanthellae [28]. Therefore, we investigated the allelopathic activities of the three most bioactive sponge species and assessed if the cytotoxic and antimicrobial properties provide them with a competitive advantage over corals.

### Allelopathic activities of sponge extracts

Extracts of *Callyspongia* sp., *Pseudoceratina* sp. and *H*. *atra*, incorporated into phytagel, had rapid negative effects on the maximum photosynthetic quantum yield of the zooxanthella that live within the coral tissue. The three sponges revealed a decrease of the photosynthetic efficiency in the corals by 19–41%. Additionally, *H*. *atra* as well as *Callyspongia* sp. also caused bleaching in branching *Porites* corals. The allelopathic effects of the sponge extracts might have been underestimated since the bioactive compounds were evenly distributed in the gel instead of concentrated on its surface. Several studies have shown that compounds identified as allelopathic agents had higher concentrations on the surface of the producing organism [57,182–184]. This is in accordance with the optimal defense theory (ODT) which states that organisms concentrate defensive compounds in parts that are especially exposed to predation or parts that are important for the fitness of an organism, e.g. reproductive organs [185–188]. Furthermore, it is likely that some reported bioactivities are slightly underestimated because non-polar compounds would not be completely extracted by using ethanol. Due to the limited laboratory conditions, ethanol was the only available solvent that could be used for the extraction of secondary metabolites. Previous studies found bioactivities often in non-polar compounds as these do not easily dilute when exuded into the surrounding seawater [189,190]. Therefore, differences which have been detected in the yield as well as bioactivity of sponge and seaweed extracts might depend on the used solvent [26,191].

The maximum photosynthetic quantum yield is a proxy for the health of the zooxanthellae, which provide corals with energy for growth and reef formation [192,193]. Thus, impairments in the health of these symbiotic algae can lead to reduced growth and less available energy that corals could invest otherwise in recruitment or spatial competition. It has already been demonstrated that corals experience a reduction in their growth, fecundity, egg size, recruits and survival during competition with algae or other benthic organisms [59,194–198].

Some coral species seem to be more susceptible to allelopathic damage. Experiments with extracts and live fragments of several algae species demonstrated that corals of the branching genera *Acropora* and *Pocillopora* were especially sensitive to allelopathic agents while corals of the massive genus *Porites* were not as much affected [183,199,200]. Some coral species differed also in their recovery potential. Corals of the genus *Porites* recovered quickly while corals of the genus *Pocillopora* showed no signs of recovery after the removal of aggressive alga individuals *in situ* [201]. Many branching corals species are already more sensitive to natural and anthropogenic disturbances and experience high mortality rates on reefs worldwide which could be further intensified by aggressive spatial competitors [183,199,201].

The ten sponge species tested in this study were previously also tested for their deterrent properties against fish predation [76]. Although, no relationship could be found between the feeding deterrence of sponge extracts and their antimicrobial activity or toxicity, the two most bioactive sponges (*H*. *atra* and *Pseudoceratina* sp.) were also defended against fish predators. For two of them their role as a successful benthic competitor was confirmed by their high abundances on the reef. *H*. *atra* was the most abundant sponge species at 5m depth, while *Pseudoceratina* sp. exhibited patchy but high abundances on the reefs around Bawe Island at 10m depth ([76]; personal observation).

## Conclusion

We demonstrated that sponges from Zanzibar possess strong growth inhibitory activities against tropical marine environmental as well as pathogenic bacterial strains. The remarkable antimicrobial activities could represent an adaptation to the high prevalence of bacteria caused by sewage outflow from Stonetown. Moreover, the cytotoxic activities and the strong allelopathic properties of *Callyspongia* sp., *H*. *atra* and *Pseudoceratina* sp. might indicate that they are important space competitors of scleractinian corals on the reef. The bioactive compounds might exert negative effects on the fecundity, reproduction or even the coral microbiome making corals even more vulnerable towards further natural or anthropogenic disturbances and pathogenesis.

The increased sewage input in combination with other global and local stressors, such as climate change, destructive fishing practices or damage to the reef through tourism activities, will most likely result in more frequently occurring sponge-coral interactions. The chemical defenses in the investigated sponges might be one reason explaining their increasing abundances from < 1% up to 7.5% on the reef at Bawe Island in recent years [8,72,76]. Therefore, the reef management around Zanzibar has to focus on mitigating anthropogenic caused disturbances such as overfishing and especially focus on the establishment of a wastewater treatment facility.

## Supporting information

S1 Fig Location of the study site Bawe Island on the western side of Zanzibar Island, Tanzania. (TIF)

S2 Fig Antimicrobial activities of the ten investigated sponge species against *Vibrio* spp.. (TIFF)

S1 Table PERMANOVA results comparing the strength of inhibition between environmental and pathogenic bacterial strains. (DOCX)

S2 Table Results of the disc diffusion assays. (XLSX)

S3 Table PERMANOVA results comparing the mortality rates of brine shrimp larvae at extract concentrations of 1000μg ml^-1^ and 100μg ml^-1^. (DOCX)

S4 Table Results of the brine shrimp assays. (XLSX)

S5 Table Effects of sponge extracts on the maximum photosynthetic quantum yield of Porites corals. (XLSX)
