# Supplementary figures and images for: Sponge chemical defenses are a possible mechanism for increasing sponge abundance on reefs in Zanzibar
Source: PLoS One. 2018 Jun 20;13(6):e0197617. doi: 10.1371/journal.pone.0197617 (PMC6010217; doi:10.1371/journal.pone.0197617)

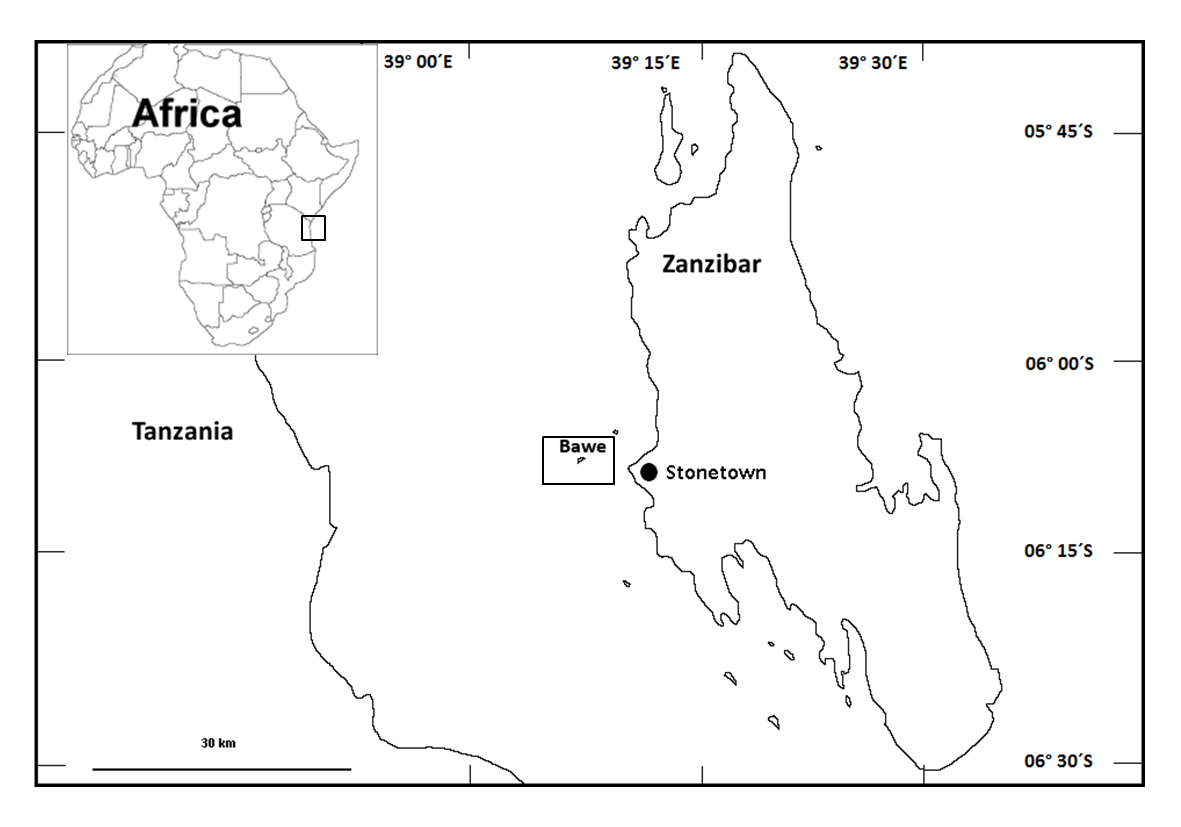

Supplement: S1 Fig — (TIF) [file pone.0197617.s001.tif]

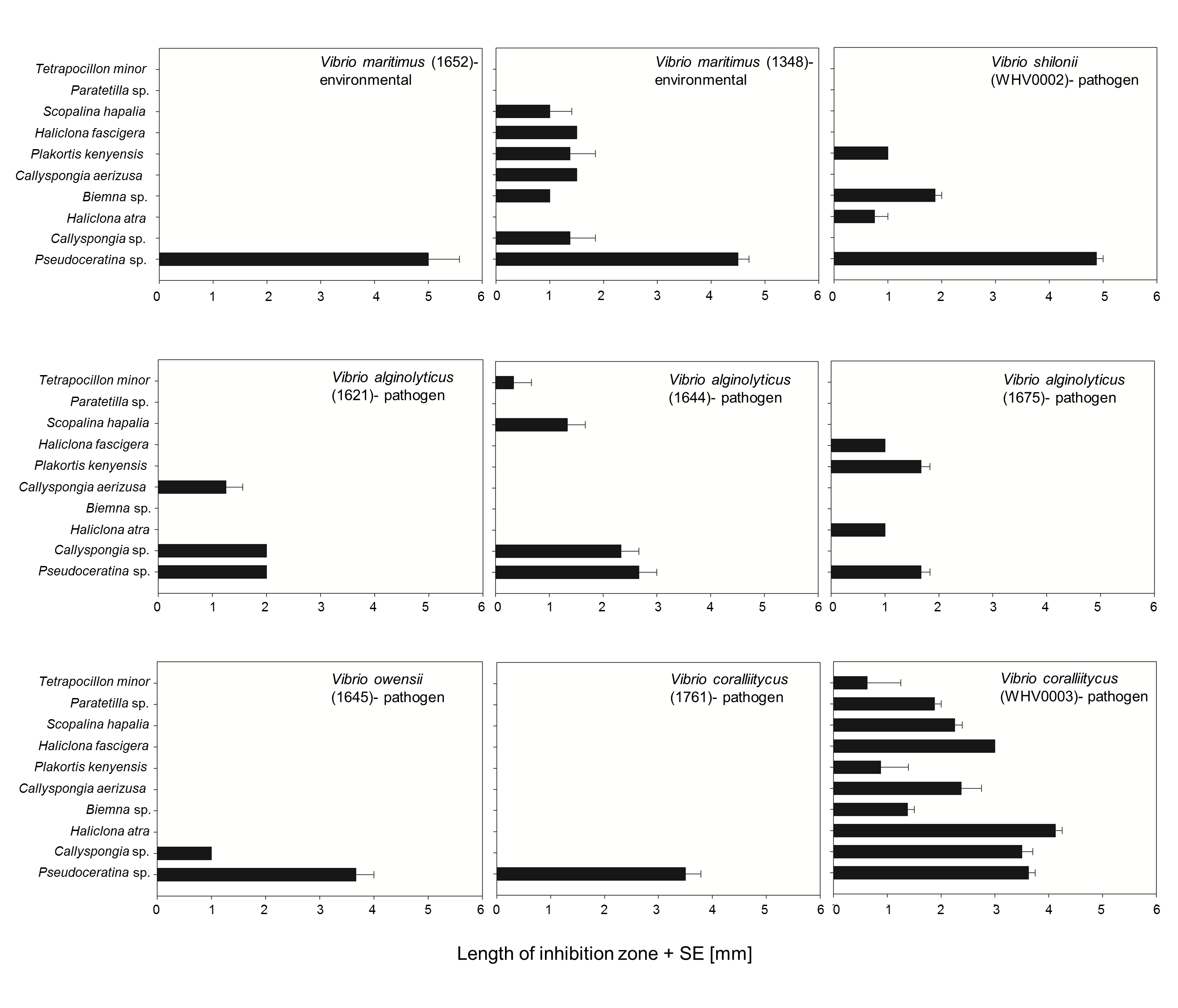

Supplement: S2 Fig — (TIFF) [file pone.0197617.s002.tiff]
