# Supplementary material for: Sponge chemical defenses are a possible mechanism for increasing sponge abundance on reefs in Zanzibar
Source: PLoS One. 2018 Jun 20;13(6):e0197617. doi: 10.1371/journal.pone.0197617 (PMC6010217; doi:10.1371/journal.pone.0197617)
Supplement: S1 Table — (DOCX) [file pone.0197617.s003.docx]

|  | df | SS | MS | Pseudo-F | p (MC) | Uniq. Per. |
| --- | --- | --- | --- | --- | --- | --- |
| *Pseudoceratina* sp. | | | | | | |
| Environ. vs. path. | 1 | 155.12 | 155.12 | 20.761 | **0.001** | 143 |
| Res | 121 | 904.7 | 7.4716 |  |  |  |
| *Callyspongia* sp. | | | | | | |
| Environ. vs. path. | 1 | 9.2418 | 9.2418 | 4.5491 | **0.036** | 77 |
| Res | 120 | 243.79 | 2.0316 |  |  |  |
| *Haliclona atra* | | | | | | |
| Environ. vs. path. | 1 | 0.0284 | 0.0284 | 0.0214 | 0.889 | 67 |
| Res | 126 | 167.28 | 1.3276 |  |  |  |
| *Biemna* sp. | | | | | | |
| Environ. vs. path. | 1 | 0.2011 | 0.2011 | 0.2390 | 0.602 | 51 |
| Res | 130 | 109.37 | 0.84127 |  |  |  |
| *Callyspongia aerizusa* | | | | | | |
| Environ. vs. path. | 1 | 0.0007 | 0.0007 | 0.0007 | 0.972 | 52 |
| Res | 115 | 110.53 | 0.96112 |  |  |  |
| *Haliclona fascigera* | | | | | | |
| Environ. vs. path. | 1 | 0.0582 | 0.0582 | 0.05986 | 0.815 | 55 |
| Res | 114 | 110.83 | 0.97217 |  |  |  |
| *Scopalina hapalia* | | | | | | |
| Environ. vs. path. | 1 | 1.1052 | 1.1052 | 1.0337 | 0.309 | 54 |
| Res | 127 | 135.78 | 1.0692 |  |  |  |
| *Plakortis kenyensis* | | | | | | |
| Environ. vs. path. | 1 | 0.0008 | 0.0008 | 0.00101 | 0.974 | 48 |
| Res | 120 | 90.231 | 0.75192 |  |  |  |
| *Paratetilla* sp. | | | | | | |
| Environ. vs. path. | 1 | 0.01033 | 0.01033 | 0.0253 | 0.892 | 34 |
| Res | 117 | 47.809 | 0.40862 |  |  |  |
| *Tetrapocillon minor* | | | | | | |
| Environ. vs. path. | 1 | 1.1111 | 1.1111 | 2.1231 | 0.164 | 25 |
| Res | 118 | 61.756 | 0.52335 |  |  |  |

environ., environmental; path., pathogenic.

Values in bold indicate p < 0.05.
