# Supplementary material for: Sponge chemical defenses are a possible mechanism for increasing sponge abundance on reefs in Zanzibar
Source: PLoS One. 2018 Jun 20;13(6):e0197617. doi: 10.1371/journal.pone.0197617 (PMC6010217; doi:10.1371/journal.pone.0197617)
Supplement: S3 Table — (DOCX) [file pone.0197617.s005.docx]

|  | Unique perms | t | p (MC) |
| --- | --- | --- | --- |
| *Pseudoceratina* sp. | | | |
| 1000μg ml^-1^ | 14 | 3.9496 | **0.003** |
| 100μg ml^-1^ | 18 | 4.088 | **0.002** |
| *Callyspongia* sp. | | | |
| 1000μg ml^-1^ |  |  | **Denominator is 0** |
| 100μg ml^-1^ |  |  | **Denominator is 0** |
| *Haliclona atra* | | | |
| 1000μg ml^-1^ | 16 | 3.2387 | **0.010** |
| 100μg ml^-1^ | 4 | 2.2064 | **0.048** |
| *Biemna* sp. |  |  |  |
| 1000μg ml^-1^ | 6 | 2.8706 | 0.016 |
| 100μg ml^-1^ | 1 | 1 | 0.347 |
| *Callyspongia aerizusa* | | | |
| 1000μg ml^-1^ | 16 | 4.1251 | **0.002** |
| 100μg ml^-1^ | 18 | 2.0793 | **0.002** |
| *Plakortis kenyensis* | | | |
| 1000μg ml^-1^ | 24 | 3.6851 | **0.005** |
| 100μg ml^-1^ | 6 | 2.0793 | 0.070 |
| *Haliclona fascigera* | | | |
| 1000μg ml^-1^ | 32 | 13.379 | **0.001** |
| 100μg ml^-1^ | 24 | 5.2453 | **0.001** |
| *Scopalina hapalia* | | | |
| 1000μg ml^-1^ | 6 | 19 | **0.001** |
| 100μg ml^-1^ | 1 | 1 | 0.343 |
| *Paratetilla* sp. | | | |
| 1000μg ml^-1^ | 4 | 2.0217 | 0.065 |
| 100μg ml^-1^ | 1 | 1 | 1 |
| *Tetrapocillon minor* | | | |
| 1000μg ml^-1^ | 4 | 2.2029 | 0.052 |
| 100μg ml^-1^ |  |  | Denominator is 0 |

Values in bold indicate p < 0.05.
